# Using evidence-based applied positive psychology to promote student well-being

**DOI:** 10.3389/fpsyg.2024.1415519

**Published:** 2024-06-26

**Authors:** Stefania Fält-Weckman, Åse Fagerlund, Monica Londen, Martin Lagerström

**Affiliations:** ^1^The Faculty of Educational Sciences, University of Helsinki, Helsinki, Finland; ^2^Folkhälsan Research Center, Helsinki, Finland; ^3^The Faculty of Arts, Psychology and Theology, Åbo Akademi University, Turku, Finland

**Keywords:** well-being, ill-being, positive psychology, positive psychology intervention, positive education

## Abstract

There has been a noticeable decline in life satisfaction among adolescents globally in recent years. The present study explored the immediate and long-term effects of a positive psychology intervention course, Study with Strength, on the well-being of students at general upper secondary school in Finland during the pandemic. Based on a partly randomized wait-list control group design, the study included a final sample of 350 students from 10 schools. Self-report measures were used to assess both immediate between-group effects and long-term within-group effects of the intervention on student well-and ill-being. A combination of methods from positive psychology and cognitive therapy were applied, and the course was administered by the participating schools’ own teachers and student-welfare personnel. The findings show that the Study with Strength intervention course enhanced the students’ experiences of positive practices at school, happiness and of positive emotions. However, the effect sizes were small. The intervention did not have any immediate effects on all aspects of well-being, negative emotions, depression, or study-related burnout. The significant, positive changes in students’ well-being in the analysis of long-term effects must be interpreted with caution. The students also reported a positive effect of the intervention both on their personal lives and in their studies. Overall, it appears that the intervention had a small but positive impact, nudging students towards enhanced well-being. The results offer valuable insights into the implementation of positive education on students at general upper secondary school aged 15–19.

## 1 Introduction

A decline in life satisfaction has been reported among adolescent students worldwide (Marquez and Long, 2021). Approximately 13 percent of the burden of disease among adolescents aged 10 to 19 could be attributed to mental disorders, affecting one in seven in this age group (World Health Organization, 2021). Recent longitudinal studies have consistently reported significant increases in psychological stress and mental-health issues among adolescents during the COVID-19 pandemic globally (Jost et al., 2023). Findings from the initial year of the pandemic indicate that globally, approximately 1 in 4 youth are experiencing clinically elevated depression symptoms, while 1 in 5 youth are experiencing clinically elevated anxiety symptoms (Racine et al., 2021). In Finland in 2021, 30% of girls aged 10–17 reported experiencing moderate to high levels of anxiety and episodes of depression lasting over 2 weeks (Aalto-Setälä et al., 2021). For boys, periods of depression were more prevalent (15%) than moderate to high levels of anxiety (8%) (Aalto-Setälä et al., 2021). In addition, many students in upper secondary education in Finland are under a lot of pressure. Admission to higher education is highly competitive in many disciplines. Students also experience social stress and peer pressure. It has been suggested, that the extensive time adolescents invest in interacting with electronic devices could be directly connected to feelings of unhappiness in that it may have replaced time that was previously dedicated to more beneficial activities (Helliwell et al., 2019).

To address the concerning development of youth mental health, the World Health Organization has emphasized the need to prioritize the mental health of adolescents as a crucial component in reaching the United Nations’ 17 Sustainable Development Goals (SDGs) (United Nations, 2015). Enhancing adolescent well-being would be an important step in promoting a healthy lifestyle and well-being among all age groups (SDG 3), and in reducing inequalities within and among countries (SDG 10). World Health Organization also supports the implementation of psychosocial interventions for all adolescents to promote positive mental health (World Health Organization, 2020). One approach to achieving this goal is to offer such interventions within the school setting, thereby ensuring that they are available to all adolescents equally.

The definition of well-being has been explored and discussed by numerous researchers (Dodge et al., 2012; Placa et al., 2013; Ruggeri et al., 2020). Keyes’ (2002) mental health continuum model describes well-being along two separate continuums: one for mental illness, and the other for mental health. The model shows how individuals may simultaneously occupy various positions on both continuums, highlighting the coexistence of the two mental states. Keyes (2002) describes the presence of mental health as flourishing and the absence of mental health as languishing. Consequently, addressing mental ill-being does not automatically enhance mental well-being. Examples of mental illness in Keyes theory include psychiatric disorders such as depression, whereas mental health requires a combination of emotional, psychological, and social well-being. Complete mental health is described by Keyes as the absence of mental illness and the presence of flourishing (Keyes, 2005). In comparison, Seligman (2011) outlines five factors that contribute to well-being, which Seligman refers to as flourishing: Positive Emotion, Engagement, Relationships, Meaning, and Accomplishment. The model is known as the PERMA model. Like Keyes (2002, 2005), Seligman argues that addressing well-being requires more than just addressing ill-being. Several interventions have been developed within the field of positive psychology to enhance well-being by addressing different aspects of these factors (Bott et al., 2017; Wingert et al., 2020; Kounenou et al., 2022).

Positive psychological interventions have shown potential as effective methods for enhancing well-being (Sin and Lyubomirsky, 2009). Positive education, as defined by White and Murray (2015), encompasses empirically validated interventions from positive psychology aimed at improving student well-being. The overarching goal is to integrate the principles of positive psychology into teaching practices and educational paradigms to foster optimal development and flourishing within the school environment (Norrish et al., 2013). It has been shown that the explicit teaching of applied positive psychology in schools enhances well-being among students (Waters, 2011; Chodkiewicz and Boyle, 2017; Tejada-Gallardo et al., 2020; Carr et al., 2023). Promoting well-being in the school environment has multiple benefits. For example, positive emotions have been shown to increase intrinsic motivation and to facilitate flexible, creative modes of thinking (Pekrun et al., 2002). After controlling for all other variables, a positive school climate together with self-efficacy and the worry component of test anxiety, predicted subjective well-being and/or grade point average (Steinmayr et al., 2018). Steinmayr et al. (2018) suggest that a positive school climate benefits student well-being as well as educational achievement. Promoting well-being among young people and protecting them from depression are associated with later academic success, thereby having both individual and national benefits (Cárdenas et al., 2022). However, even though several studies have reported a connection between student well-being and academic achievement, the findings have shown some inconsistency (Bücker et al., 2018; Clarke, 2020). Nonetheless, there does seem to be some evidence of a positive relationship between student well-being and academic achievement (Klapp et al., 2023).

Despite the promising results, further research on positive education is needed to establish best practices in positive psychology interventions in schools (Chodkiewicz and Boyle, 2017). Chodkiewicz and Boyle (2017) emphasize the need to initiate investment in high-quality teacher training and support. There has also been a demand for larger sample sizes in research on positive-psychology intervention to avoid small sample size bias (White et al., 2019). Sample sizes of the studies included in the meta-analysis by White et al. (2019) reanalyzing the meta-analyses of Sin and Lyubomirsky (2009) and Bolier et al. (2013), varied from 12 to 208 participants. Additionally, there is a significant demand for long-term or longitudinal study designs (van Zyl et al., 2019).

The aim of the present study was to examine the efficacy of Study with Strength, a positive-psychology intervention course, in enhancing upper secondary school student well-being in Finland short-and long-term. The aim was also to contribute with practical insights regarding the implementation of a positive psychology intervention in upper secondary education through intensive training and support for the participating teachers. We also aimed to collaborate with the teachers in developing the intervention. The following research questions were addressed: (1) what are the immediate effects of the positive psychology intervention course, Study with Strength, on students’ experiences of their well-and ill-being? (2) What are the long-term effects?

We hypothesized that, after the intervention, participants in the experimental group would show higher levels of well-being at school and in their everyday lives than those in the control group. Even though we expected the intervention to have a positive impact mainly on well-being, we also hypothesized that the experimental group would experience less ill-being at school and in their everyday lives after the intervention compared to the control group. We further posited that the effects of the intervention would be not only short-term (immediately following the intervention) but also long-term (extending until the end of the school year).

## 2 Materials and methods

### 2.1 Design and participants

Finland is a bilingual country with Finnish and Swedish as the two national languages. Although the majority of the population are native Finnish speakers, there are parallel school systems in Finnish and Swedish. Eleven Swedish-language general upper secondary schools in Finland took part in the study, chosen by means of convenience sampling. Information about the project was given at a national meeting for principals at general upper secondary schools organized by the Finnish National Agency for Education, after which those who were interested could then sign up. Ten of the eleven schools participating in the project signed up for the study after the meeting. In addition, the research group presented the project to one school, but it was dropped from the analysis because it was the only school that could not arrange a control group and only had two students who were interested in participating in the study. The initial sample comprised 419 students. However, 67 students withdrew from the study before the first measurement was taken, or did not submit a single measurement. Their reasons for withdrawal included having changed their mind about taking the intervention course, time constraints, and that they were under too much stress to commit to the study. The final sample included 350 students.

**Figure 1:**
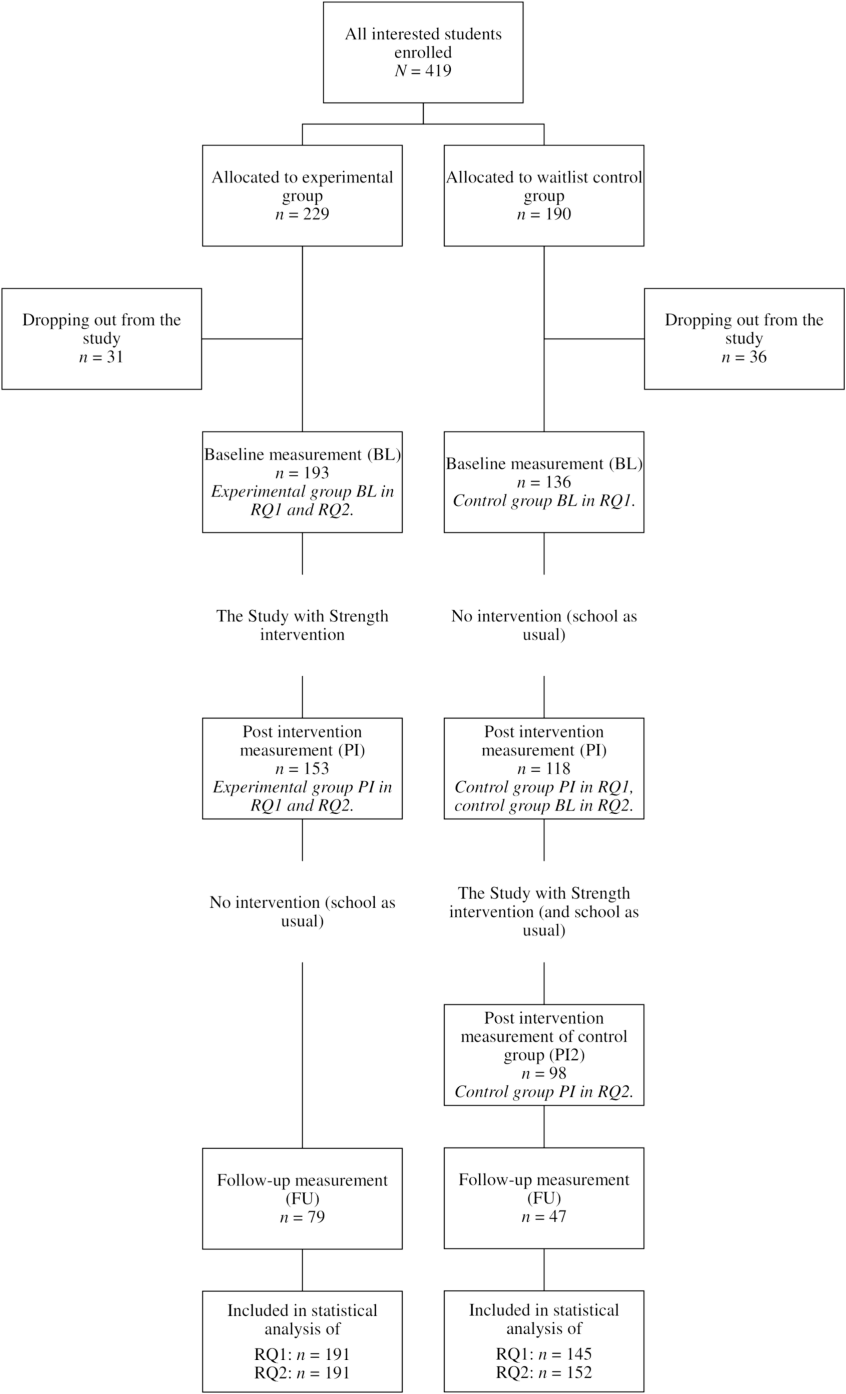
Flow diagram of the study design and the numbers of included participants throughout the study.

General upper secondary education typically spans three to 4 years following basic education. Most of the students were 15–19 years old. Written consent was obtained from them before the study. They had all turned 15 earlier, thus parental consent was not required according to Finnish legislation. However, the parents of the students taking the course were informed about the study. Table 1 shows the demographic profiles of the participants included in the analysis. Figure 1 presents the study design and the numbers of included participants throughout the study.

**Table 1:** Demographic characteristics of the participants included in the analysis.

Baseline charasteristic	All participants, *n* = 343		Intervention group, *n* = 191		Control group, *n* = 152	
	*n*	%	*n*	%	*n*	%
Female	254	74.1	149	78.0	105	69.1
Male	63	18.4	35	18.3	28	18.4
Other	2	0.6	2	1.0	0	0.0
Missing	24	7.0	5	2.6	19	12.5
First-year students (15–16 years old)	122	35.6	66	34.6	56	36.8
Second-year students (16–17 years old)	113	32.9	61	31.9	52	34.2
Third-year students (17–18 years old)	84	24.5	58	30.4	26	17.1
Students from the fourth year onwards (18 years or older)	1	0.3	1	0.5	0	0.0
Missing	23	6.7	5	2.6	18	11.8

Demographic characteristics were self-reported only during the initial baseline assessment. Charasteristics of students who did not complete the baseline measurement are reported as “missing.”

The study was based on a randomized waitlist control group design. All the students who volunteered to participate were assigned to an experimental group or a waitlist control group. However, the randomization of all participants was not possible in some schools for curriculum-related reasons. Of the ten schools included in the analysis, four were able to complete the randomization (*n* = 112), two were able to randomize around half of their participants (*n* = 106), one was able to complete a cluster randomization (*n* = 62), and three could not randomize their participants at all (*n* = 70). The reason for choosing the waitlist control group design, was to ensure that all students had equal opportunities to participate in the intervention. Students in the control group continued with their regular studies while on the waitlist. The intervention was organized during the same school year for the control group to ensure equal possibilities for students in their final year of studies. Due to this, the control group students received the intervention before or during the follow-up at the end of the school year and could no longer be used as a control group for the follow-up measurement. It was also possible to take the course without participating in the study.

Data were collected during the school years 2020–2021 (eight schools included in the analysis) and 2021–2022 (two schools included). The schools were in different cities located in Swedish-speaking regions of Finland: five in southern Finland, four in Ostrobothnia and one on the Åland Islands. Between 11 and 62 students from each school participated in the study (*M* = 35.10, *SD* = 16.99, *Mdn* = 33.50). The course was optional in all except one of the schools, in which it was compulsory for all first-year students (*n* = 62). Nine schools completed two courses, one school completed four and one completed seven. In each school, the group sizes varied from under ten students to over twenty students.

### 2.2 Procedure

The students in the experimental group were given a minimum of 30 min to complete the measurement, namely a set of questionnaires distributed during the first (baseline, BL) and last (post-intervention, PI) lesson on the intervention course. The questionnaires were online, thus the students could continue with them later. Those in the waitlist control group received them at the same time as those in the experimental group and filled them in during their spare time. There was no specific deadline for completing the questionnaires, but email reminders were sent to students who did not complete the questionnaire within a week. Since the participating schools did not organize all courses simultaneously, the baseline and the post intervention questionnaires had to be left open until the final measurements were done for all schools. For example, during the first year of the data Research Topic, since the first school started with the experiment group in September, and the last school in December, the baseline questionnaire had to be accessible for altogether around 5 months.

A third measurement (second post-intervention, PI2) was organized for the students in the waitlist control group after they had completed the course. Students were allocated time to fill out the questionnaires during the last lesson of the intervention course. Finally, a shorter follow-up measurement (FU) was organized at the end of the school year for students in the experimental group and the control group, approximately 1–6 months after completing the intervention course. Those in the control group who completed the intervention course at the end of the school year did not take part in the follow-up measurement because it would have overlapped with their second post-intervention measurement (*n* = 41). Students in both groups who completed the baseline questionnaire 2 weeks or less before the post-intervention questionnaire were excluded from the analysis (*n* = 7).

The study was approved by the University of Helsinki Ethical Review Board in the Humanities and Social and Behavioural Sciences in January 2020. Research permission was granted from all the schools and municipalities participating in the study.

### 2.3 The study with strength intervention

The Study with Strength project was initiated following numerous requests from teachers and principals in upper secondary schools. They were concerned about the well-being of their students and were inspired by the positive outcome of the predecessor intervention project “Strength, Happiness and Compassion” for students aged 7–12 (Laakso et al., 2021, 2022, 2023).

Study with Strength was developed by the research group in close collaboration with the participating schools in both format and content, to suit the needs of the schools while closely following scientific methods and ethical guidelines. The course contents reflect evidence-based methods from applied positive psychology, cognitive behavioral therapy, and mindfulness, (see Table 2 for an overview of the lesson outlines) and are built on the PERMA-theory of well-being (Seligman, 2011). The aim is to enhance the well-being and mental resources of students. The course combines theory and practice, and is designed to encourage students to engage in active participation and experiential learning. In its design it follows the Finnish National core curriculum (Finnish National Agency for Education, 2019) in terms of duration, which made it possible for students to earn credits for completing it. No submission work or tests were included.

**Table 2:** Lesson outlines for the Study with Strength intervention course.

Lesson	Outline	Homework
1.Introduction	Research questionnaire Introduction to positive psychology (Seligman, 2011) Exercise from the Appreciative Inquiry (AI) model (Cooperrider and Whitney, 2005)	Gratefulness diary for 2 weeks
2.Character strengths	Character strengths work (Peterson and Seligman, 2004; Niemiec, 2014)	Strength spotting
3.Mindfulness	Introduction to mindfulness (Kabat-Zinn, 2013) Raisin and body scan meditation	Informal and formal mindfulness practice
4.Prioritizing values	Mindful breathing Prioritizing values Exercise from the AI (Cooperrider and Whitney, 2005)	Monitoring time management
5.Meaningful goal setting	Mindful phone usage exercise Exercise from the AI (Cooperrider and Whitney, 2005) Meaningful goal setting	The first step of the meaningful goal
6.Character strengths continued	Over-and under-use of character strengths (Niemiec, 2014) Mindful breathing/body scan meditation	Character strengths 360° (Niemiec, 2014)
7.Positive emotions	Broaden-and-build theory of positive emotions and savoring (Fredrickson, 2013a) Mindful breathing	Savoring exercise
8.Positive relationships	Body scan meditation Active-Constructive Responding	Response style observing
9.Strengths and goal setting	Best possible self exercise (Niemiec, 2014) Character strengths in goal setting	Use a character strength in new ways for a week (Niemiec, 2014)
10.Mindset	Mindset and neuroplasticity (Dweck, 2007) Mindful breathing/body scan	No homework
11.Mindset continued	Mindful breathing Mindset	No homework
12.Self-efficacy and flow	Self-efficacy (Bandura, 1977) Flow (Csikszentmihalyi, 2008)	Flow exercise
13.Thought patterns	Introduction to the cognitive model (Beck, 2020) Mindfulness of thoughts	Thought monitoring
14.Resilience and cognitive flexibility	Mindful breathing Resilience Cognitive distortions (Beck, 2020)	Reflection about cognitive distortions
15.Conflict management	Mindfulness exercise for challenging situations Assertiveness	Reflection about assertiveness
16.Positive emotions and self-compassion	Theory and exercises (Fredrickson, 2013a; Neff, 2023)	Reflection about positivity ratio
17.Closure	Research questionnaire Repetition Loving kindness meditation	

The course comprises 17 lessons, each lasting 75 min. The lessons include theoretical input and exercises for both individuals and small groups, mindfulness meditation, discussions in groups and pairs, and suggestions for homework to consolidate the practices. The schools were allowed to make small adjustments in the length of the intervention so they could implement it in the best possible way. The lessons were distributed over approximately 2–3 months in nine of the ten schools, with 2–3 lessons per week. In the remaining school (*n* = 19) it ran for almost 4 months, with just one lesson per week.

The course administrators included the participating schools’ own teachers, school psychologists and social workers, guidance counsellors, and principals, who were all invited to take part in the training. In 2020, five full in-service training days were organized for the first nine participating schools’ teams to train them thoroughly in teaching the material. A further aim with the training days was to give the staff the opportunity to try out the intervention themselves before administering it to their students: the staff did the same exercises during the training days as the students during the intervention course. The staff was also encouraged to do the same homework as the students. The first training day was attended in person, but due to the Covid-19 pandemic the remaining 4 days were organized online. Similar training days were organized for teams from the additional two schools participating during school year 2021–2022. Completing the in-service training was a prerequisite for administering the course.

To ensure intervention consistency across schools, the teachers were provided with all the necessary material, which was designed to suit both in-class and online teaching because of the pandemic. The material for each lesson included detailed outlines, as well as presentations with notes explaining the theory and the activities. As part of the project, all students taking the course were provided with a notebook that they were encouraged to use for writing down their reflections during the course. The aim was to inspire them to record their most helpful insights in the notebook for further reflection and future use. The project group stayed in contact with the schools during the whole project, and members of the research group were available for counselling when needed.

## 3 Measures

Five self-report measures were used to assess different aspects of the participants’ well-and ill-being. In addition, four questions concerned the perceived effects of the intervention. All of these were used for the baseline and post-intervention measurements. The follow-up measure was shortened to encourage as many participants as possible to complete the final survey, and included only one aspect of well-being (The EPOCH Measure of Adolescent Well-being) and one of ill-being (The Student Burnout Inventory).

### 3.1 Well-being

The EPOCH Measure of Adolescent Well-being (Kern et al., 2015), which is based on PERMA-theory and was created specifically for adolescents, was used to measure general well-being. The EPOCH is a self-report questionnaire consisting of 20 statements divided among five subscales: Engagement, Perseverance, Optimism, Connectedness, and Happiness. The participants were asked to rate the extent to which each statement described them (e.g., “I am optimistic about my future”) on a 5-point Likert scale ranging from 1 (almost never) to 5 (almost always). The Cronbach’s α of the subscales and the total score ranged from 0.70 to 0.89.

An adapted version of the Positive Practices Survey (PPS) was used to measure well-being at school (Cameron et al., 2011). The PPS was developed to measure “positively deviant, affirming, and virtuous practices” in an organizational context (Cameron et al., 2011, p. 7). The Study with Strength research group adapted the survey to measure positive practices and well-being at school. This involved replacing organizational and work-related words to match the school environment and studying on the upper-secondary level: e.g., “We care for fellow employees who are struggling” was changed to “We care for fellow students who are struggling.” The survey consists of 29 items divided among six subscales: Dignity, Support, Care, Meaning, Inspiration, and Forgiveness. Participants were asked to think about their days at school as they rated the extent to which they could relate to each statement (e.g., “we communicate the good we see in one another”) on a 5-point Likert scale, ranging from 1 (completely disagree) to 5 (completely agree). The Cronbach’s α of the subscales and the total score ranged from 0.85 to 0.96.

### 3.2 Positive and negative affect

Positive and negative affect, which are aspects of both well-and ill-being, were measured using the Positive and Negative Affect Schedule (PANAS) (Watson et al., 1988; Crawford and Henry, 2004). PANAS is a self-report questionnaire consisting of two 10-item subscales: Positive affect and Negative affect. The items are single-word adjectives representing positive (e.g., “inspired,” “enthusiastic”) or negative (e.g., “scared,” “distressed”) affect. Participants were asked to rate the extent to which they had experienced each one during the past week on a 5-point Likert scale, ranging from 1 (very slightly or not at all) to 5 (extremely much). The Cronbach’s α was 0.87 for both Positive and Negative affect. Calculation of a Positivity ratio, in other words the ratio between Positive and Negative affect, involved dividing the mean score for Positive affect by the mean score for Negative affect. A higher Positivity ratio indicates more positivity than negativity (Fredrickson, 2013b).

### 3.3 Ill-being

General ill-being was measured on the Centre for Epidemiologic Studies Depression Scale (CES-D) (Radloff, 1977), which is a widely used self-report questionnaire consisting of 20 statements that is intended to measure the frequency and severity of depressive symptoms. A four-factor structure (Depressed affect, Positive affect, Somatic symptoms, and Interpersonal difficulties) was initially identified in adults, replicated later with adolescents (Blodgett et al., 2021). Each of the four factors includes between two and seven statements (e.g., “I thought my life had been a failure”). In completing the questionnaire the participants were asked to think about their past week, and to rate how often they had felt the way the statements indicated on a 4-point Likert scale ranging from 1 (rarely or none of the time) to 4 (most or all the time). The Cronbach’s α of the four different factors and the total score ranged from 0.65 to 0.91.

Ill-being related to studies was measured on the Student Burnout Inventory (SBI) (Salmela-Aro et al., 2009), which was developed to measure school burnout among students in upper secondary education. The inventory consists of ten statements, divided among the three factors of school burnout: exhaustion at school, cynicism towards the meaning of school, and a sense of inadequacy at school. The participants were asked to think about their situation (estimated from the previous month) as they rated the extent to which they could relate to each statement on a 5-point Likert scale ranging from 1 (completely disagree) to 5 (agree). The Cronbach’s α of the subscales and the total score ranged from 0.69 to 0.85.

### 3.4 Perceived effects of the intervention

After completing the Study with Strength intervention, members of both the experimental group and the waitlist control group were asked whether they would recommend the course to other students (yes or no). They were also asked to rate the usefulness of the content for their studies and for their personal life on a 5-point Likert scale ranging from 1 (not at all useful) to 5 (very useful). Finally, they were asked how the course had affected their well-being on a 4-point Likert scale (1 = in a very positive way, 2 = in a positive way, 3 = cannot say, 4 = in a negative way).

## 4 Results

IBM’s SPSS Statistics 27 was used to process and analyze the survey data and thereby to assess the effects of the Study with Strength intervention course. Linear mixed-effects models were then applied to examine both the immediate and the long-term effects of the intervention on the outcome variables. All the models were estimated with random intercepts based on restricted maximum likelihood (REML). Measurement times were nested within participants and group, time, and gender in the analysis of the immediate effects of the intervention, and the interaction between the group and the measurement time were computed as fixed effects. The analysis of long-term effects did not include measurement of the group, or the interaction between group and the measurement time as fixed effects.

### 4.1 The immediate effects of the intervention

The analysis related to the first research question (immediate effects measured directly after the intervention) involved the inclusion of a waitlist control group. The intraclass correlation (ICC) ranged from 0.52 to 0.80 for all 24 analyses, indicating that the participants had highly varying scores at the baseline measure. Table 3 shows the estimated marginal means for the groups and the interaction effects from the models.

**Table 3:** Estimated marginal means for the groups and the inLteraction effects from the immediate effect models.

Measure	Baseline (BL)				Post-intervention (PI)				Time × Group	Cohen’s *d*
	Experimental *n* = 186		Control *n* = 134		Experimental *n* = 150		Control *n* = 117		*F*(df1, df2), *p*	
	*M*	*SE*	*M*	*SE*	*M*	*SE*	*M*	*SE*		
EPOCH	3.10	0.13	3.14	0.14	3.22	0.13	3.17	0.14	*F*(1,267.89) = 3.46, *p* = 0.064	0.10
Engagement (EPOCH)	2.59	0.17	2.62	0.18	2.81	0.17	2.73	0.18	*F*(1,279.00) = 1.94, *p* = 0.165	0.12
Perseverance (EPOCH)	3.37	0.17	3.46	0.17	3.36	0.17	3.44	0.17	*F*(1,263.86) = 0.07, *p* = 0.798	0.02
Optimism (EPOCH)	3.03	0.18	3.09	0.19	3.18	0.18	3.15	0.19	*F*(1,264.44) = 1.39, *p* = 0.240	0.07
Connectedness (EPOCH)	3.94	0.17	3.90	0.18	3.93	0.17	3.90	0.18	*F*(1,265.08) = 0.67, *p* = 0.796	0.02
Happiness (EPOCH)	3.08	0.20	3.15	0.21	3.22	0.20	3.11	0.21	*F*(1,263.79) = 7.81, *p* = 0.006^*^	0.18
Positive practices survey (PPS)	3.24	0.15	3.21	0.16	3.39	0.15	3.19	0.16	*F*(1,267.98) = 7.74, *p* = 0.006^*^	0.21
Dignity (PPS)	3.49	0.17	3.45	0.18	3.60	0.17	3.44	0.18	*F*(1,269.73) = 2.73, *p* = 0.100	0.14
Support (PPS)	3.36	0.17	3.25	0.17	3.48	0.17	3.23	0.18	*F*(1,274.64) = 3.58, *p* = 0.060	0.16
Caring (PPS)	3.09	0.20	3.04	0.21	3.28	0.20	3.06	0.21	*F*(1,271.41) = 3.30, *p* = 0.070	0.15
Meaning (PPS)	2.91	0.17	2.99	0.18	3.10	0.17	2.95	0.18	*F*(1,272.37) = 7.84, *p* = 0.005^*^	0.24
Inspiration (PPS)	2.98	0.19	2.95	0.20	3.19	0.19	2.94	0.20	*F*(1,276.94) = 5.34, *p* = 0.022^*^	0.24
Forgiveness (PPS)	3.35	0.20	3.36	0.21	3.53	0.20	3.30	0.21	*F*(1,278.78) = 5.20, *p* = 0.023^*^	0.26
PANAS positive emotions	2.77	0.14	2.85	0.15	2.93	0.15	2.80	0.15	*F*(1,272.41) = 8.19, *p* = 0.005^*^	0.29
PANAS negative emotions	2.79	0.17	2.77	0.18	2.71	0.17	2.68	0.18	*F*(1,268.04) = 0.01, *p* = 0.929	0.01
PANAS ratio (positive:negative)	1.17	0.13	1.18	0.14	1.29	0.13	1.22	0.14	*F*(1,269.52) = 1.39, *p* = 0.240	0.09
CES-D	1.28	0.13	1.22	0.14	1.23	0.13	1.23	0.14	*F*(1,263.43) = 1.66, *p* = 0.199	−0.08
Depressed affect (CES-D)	1.21	0.16	1.13	0.17	1.15	0.16	1.13	0.17	*F*(1,265.91) = 0.54, *p* = 0.464	−0.05
Positive affect (CES-D)	1.51	0.15	1.43	0.16	1.32	0.15	1.42	0.16	*F*(1,264.45) = 6.96, *p* = 0.009^*^	−0.18
Somatic complaints (CES-D)	1.33	0.14	1.26	0.14	1.32	0.14	1.30	0.14	*F*(1,268.48) = 0.50, *p* = 0.480	−0.05
Interpersonal problems (CES-D)	0.96	0.16	0.99	0.17	1.00	0.16	1.01	0.17	*F*(1,275.82) = 0.10, *p* = 0.919	−0.01
Student burnout inventory (SBI)	3.44	0.22	3.38	0.23	3.60	0.22	3.58	0.23	*F*(1,268.75) = 0.86, *p* = 0.354	−0.08
Exhaustion (SBI)	3.65	0.23	3.66	0.24	3.68	0.23	3.80	0.24	*F*(1,272.23) = 1.25, *p* = 0.265	−0.12
Cynicism (SBI)	3.09	0.30	3.02	0.32	3.38	0.30	3.25	0.32	*F*(1,266.23) = 0.29, *p* = 0.589	0.08
Inadequacy (SBI)	3.50	0.27	3.35	0.28	3.56	0.27	3.61	0.28	*F*(1,271.12) = 2.42, *p* = 0.121	−0.18

M stands for the estimated marginal mean. ^*^Significant interaction at the 0.05 level. Cohen’s *d* was calculated with raw means using the formula: [(M_Experimental, PI_ − M_Experimental, BL_) − (M_Control, PI_ − M_Control, BL_)]/SD_BL, pooled_ where SD_BL, pooled_ was calculated as $\sqrt{\left(SD_{BL,\mathrm{Experiment}}+SD_{BL,\mathrm{Control}}\right)/2}\text{.}$

As predicted, there was a significant interaction effect between time and group in positive practices at school based on the PPS measure. Significant interaction effects between time and group were also visible in the PPS subscales Meaning, Inspiration and Forgiveness, but not in Dignity, Support, or Caring. This indicates that the students in the intervention group experienced more positive practices at school, especially related to meaning, inspiration, and forgiveness, than those in the waitlist control group. However, the effect sizes of the significant interactions were small (*d* = 0.21–0.26).

A significant interaction effect between time and group was also evident in positive emotions measured on the PANAS scale (*d* = 0.29), although there were no significant interaction effects between time and group in negative emotions similarly measured. In contrast to our expectations, there were no significant interaction effects between time and group regarding the positivity ratio, either. This indicates that the small increase in experiences of positive emotions among members of the intervention group as an immediate effect of the intervention, compared to the control group, was not big enough to change the ratio of positive and negative emotions.

Although students in the intervention group reported an increase in different aspects of their well-being immediately after the intervention, both at school (PPS) and in everyday life (PANAS), no interaction effect between time and group was evident in the total EPOCH score. However, a small significant interaction effect (*d* = 0.18) was visible on the Happiness subscale, indicating a small increase in the experience of happiness in everyday life as an immediate effect of the intervention among members of the intervention group compared to the control group.

There were no significant interaction effects between time and group on the CES-D and the SBI total scores, indicating the absence of significant differences in the development of ill-being between the intervention group and the experimental group during the intervention. Similarly, no interaction effects were observed on the CES-D and SBI subscales, except for a positive affect (*d* = −0.18) on the former.

There were significant main effects of gender on all the scales except the EPOCH subscales Engagement, Positive emotions and Happiness, the PPS subscale Forgiveness, and the SBI subscale Cynicism. Female students, on average, had lower scores on the well-being measures (PPS, EPOCH, Positive emotions), except for the EPOCH subscale Connectedness. Female students also had higher average scores than male students on ill-being measures (Negative emotions, CES-D, SBI).

### 4.2 The long-term effects of the intervention

The analysis of the second research question (long-term effects measured at the end of the school year) did not include a control group. Instead, data from the experimental group were combined with that of the control group, which had also taken the intervention course having been on the waitlist. Figure 1 presents a flow diagram of the study design. The intraclass correlation (ICC) ranged from 0.59 to 0.74 for all 10 analyses, indicating in the case of RQ1 that the participants had highly varied scores at the baseline measure. Table 4 shows the estimated marginal means for the groups and the interaction effects from the models. Long-term effects were determined as a significant change from baseline to post-intervention and a non-significant change from post-intervention to follow-up.

**Table 4:** Estimated marginal means for the groups and the interaction effects from the long-term effect models.

Measure	Baseline (BL) *n* = 303		Post-intervention (PI) *n* = 247		Follow-up (FU) *n* = 124		Time	Cohen’s *d*		
	*M*	*SE*	*M*	*SE*	*M*	*SE*	*p*	BL-PI	PI-FU	BL-FU
EPOCH total	3.11	0.13	3.22	0.13	3.14	0.13	<0.001^***^	0.19^*^	−0.10	0.09
Engagement (EPOCH)	2.60	0.17	2.89	0.17	2.66	0.17	<0.001^***^	0.36^*^	−0.24^*^	0.12
Perseverance (EPOCH)	3.36	0.16	3.39	0.16	3.31	0.16	0.373	−0.07	−0.01	−0.08
Optimism (EPOCH)	2.99	0.17	3.30	0.17	3.11	0.17	<0.001^***^	0.42^*^	−0.23^*^	0.19
Connectedness (EPOCH)	3.97	0.17	3.66	0.17	3.91	0.17	<0.001^***^	−0.37^*^	0.32^*^	−0.06
Happiness (EPOCH)	3.08	0.19	3.15	0.19	3.12	0.19	0.254	0.13	−0.05	0.08
Student burnout inventory (SBI)	3.47	0.22	3.51	0.22	3.51	0.22	0.739	0.01	0.01	0.02
Exhaustion (SBI)	3.71	0.23	3.70	0.23	3.62	0.23	0.539	−0.00	−0.06	−0.06
Cynicism (SBI)	3.10	0.30	3.24	0.30	3.33	0.30	0.019^*^	0.04	0.08	0.12^*^
Inadequacy (SBI)	3.54	0.30	3.54	0.30	3.54	0.30	0.998	−0.01	0.01	0.00

M stands for the estimated marginal mean.^*^Significant mean difference at the 0.05 level, ^***^Significant mean difference at the 0.001 level. Cohen’s *d* was calculated with raw means using the formula: (M_Timepoint 2_ − M_Timepoint 1_)/SD_BL_.

There were significant main effects of time on the total EPOCH score, and the subscales Engagement, Optimism and Connectedness, as well as the SBI subscale Cynisism. From baseline to post-intervention, there were significant positive effects on the total EPOCH score and the subscales Engagement and Optimism (*d* = 0.19–0.42). There was also a significant, negative effect on the Connectedness subscale between these measurement timepoints (*d* = −0.37). These results indicate that there was a positive effect on the participants’ general well-being during the intervention, and especially on their experiences of engagement and optimism. The findings further indicate a negative effect on experiences of connectedness. However, they must be interpreted with caution given the lack of a control group and the differences in measurement timepoints among the participants, and therefore should not be held comparable with the results from the analysis of the immediate effects of the intervention.

Although there was a significant decline in the positive effects on Engagement (*d* = −0.24) and Optimism (*d* = −0.23) between post-intervention and follow-up, the decline in the total score of the EPOCH was not significant. There was also a significant positive effect on the Connectedness subscale (*d* = 0.32) between these measurement timepoints, indicating a positive effect on the experience of connectedness after the intervention. Lastly, there was a significant positive effect from baseline to follow-up on the SBI Cynicism subscale (*d* = 0.12), indicating an increase in cynicism during the whole measurement period.

There were significant main effects of gender on the total EPOCH score and on the subscales Optimism and Happiness, as well as on the total SBI score and all the subscales except Cynicism. Compared to the male students, the female students, on average, had lower EPOCH scores except on the Connectedness subscale, and higher scores on the SBI.

### 4.3 The perceived effects of the intervention

Most participants (92.6%) would recommend the course to other students. It was perceived as more beneficial for one’s personal life (*M* = 3.82, *SD* = 0.92) than for studies (*M* = 3.56, *SD* = 0.94). Among the students, 7.8% reported a very positive impact on well-being, and 58.2% a positive impact. Two students (0.8%) reported a negative impact, and 33.2% of the students could not say what the effects of the course on their well-being were.

## 5 Discussion

Our aim in the current study was to explore the immediate and long-term effects of the PP intervention Study with Strength course on the well-and ill-being of students at upper secondary school. The analysis of the immediate effects included a control group, but due to the waitlist design of the study that of the long-term effects did not. The results from the analysis of the immediate effects indicate that the intervention had a small positive effect on the participants’ well-being at school. The intervention also had a small, positive immediate effect on the participants’ happiness and positive emotions in everyday life. However, there were no effects on symptoms of depression or study-related burnout. According to the results from the analysis of long-term effects, in turn, there was a small increase in the participants’ general well-being during the intervention, that did not decline significantly from post intervention to follow-up. However, there was also a decrease in experiencing connectedness during the intervention, and a small increase in cynicism during the whole measurement period from baseline to follow-up.

Overall, the findings indicate that the Study with Strength intervention does enhance specific aspects of student well-being, including positive practices at school, positive emotions, and happiness. However, it did not affect all aspects of general well-being. Neither did it diminish student ill-being, including symptoms of depression and study-related burnout. The dimensional view of mental health introduced by Corey Keyes (2002) might shed light on these findings. According to Keyes, mental health and mental illness may co-exist, meaning that manipulating either well-or ill-being does not necessarily have an impact on the other. Despite this, the question persists as to why the intervention failed to affect student ill-being, given that positive psychology interventions have previously demonstrated efficacy, for instance, in alleviating symptoms of depression (Carr et al., 2023). However, well-being has also been described as the balance point between an individual’s resource pool and the challenges they face (Dodge et al., 2012). Interventions that strengthen individuals’ perceptions of their resources, such as strengths and positive relationships, are therefore vital. According to the results, the intervention course might have functioned as an opportunity to cultivate positive emotions, which could be considered a solid pathway on which to attain psychological growth and enhance both psychological and physical well-being over time (Fredrickson, 2004).

The change in positive emotions further suggests that the intervention might have influenced participants’ experiences of at least one of the factors in the PERMA model of well-being (Seligman, 2011), upon which the intervention was based. Additionally, the analysis of positive practices at school reveals potential impact on other factors: engagement (the increase of inspiration), relationships (the increase of forgiveness), and meaning (the increase of meaning). According to these results it seems that the intervention was better suited to strengthening the participants’ well-being at school rather than in their lives in general. One possible explanation for this is that the study was conducted in schools, and thus a significant portion of the intervention content was tailored to the school environment. This was done by providing the students with examples of how to use different well-being skills in school-related contexts, and allowing them to practice them on their own and with each other. However, the participants perceived the course as more beneficial for their personal life than for their studies. This discrepancy could reflect how the participants understood the question: they might have been evaluating the impact of the course on factors such as academic success, rather than school-related well-being.

Even though we found evidence of a positive impact of the intervention, the effect sizes were small in both the immediate and the long-term measurements. Unfortunately, the results might have been strongly affected by the COVID-19 pandemic, in that most of the training of intervention leaders was conducted online, as were most of the experimental group courses during autumn 2020. Delivering the intervention online may have been less effective than in the classroom (West, 2023), especially given that it was originally planned for contact teaching and then adapted for online teaching. The pandemic might also explain the decline in experiences of connectedness observed from baseline to post-intervention measurements in the analysis of the intervention’s long-term effects: the time span between the baseline and the post-intervention measurements was from the autumn of 2020 to the early months of 2021, a period when many schools were either partially or entirely engaged in online teaching. The findings of differences in well-and ill-being between genders also parallel those observed in Finland during the pandemic, where girls exhibited higher levels of depression compared to boys (Aalto-Setälä et al., 2021).

Regarding the long-term effects of the intervention, the results are directive in that there was no control group for the follow-up measurement. There may also have been a difference between the development of well-and ill-being in the experimental group and the control group attributable to the study setting: students in the former group generally had more time between the post-intervention measurement and the follow-up measurement than their counterparts in the control group, and this could have confounded the results of the long-term analysis. Moreover, the teachers might have become more skilled in teaching the intervention course, leading to better outcomes when it was implemented with the control group.

Despite the small effect sizes, however, most of the students reported that the course had a positive impact on their well-being: 92.7% of the participants would have recommended it to other students. There are many possible explanations of these results. The well-being measurements we used may not have been the most suitable for describing changes triggered by the intervention. In addition, students’ perceptions of its benefits might have been affected by the time they invested in it. They may also have experienced benefits even from the small changes. It is worth pointing out that 33.2% of the participants were not sure whether the intervention had a positive impact on their well-being: they might not have experienced strong effects, or they may have been cautious about attributing any effects on loose grounds. Additionally, two participants mentioned experiencing negative effects on their well-being. Unfortunately, the reasons for this remain unknown. Possible explanations may be that the course was perceived as excessively time-consuming, or as incorporating themes that made these students feel uncomfortable.

### 5.1 Strengths and limitations

The foremost strength of this study is its partly randomized waitlist control-group design in its exploration of the immediate effects of the intervention in a real-world educational context. A limitation of the design is that the randomization of all participants was not possible for reasons related to the students’ curricula. Had it been possible it may have attracted students with different interests to the experimental group and the control group. However, the groups were checked for differences at baseline, and none were found. Regarding the use of a waitlist control group, research findings indicate that being placed on a waitlist may lead to a reduction in symptoms due to treatment expectancy effects, for example (Devilly and McFarlane, 2009; Hesser et al., 2011). This could have weakened the observed effects of the intervention.

Another strength of the study is the participation of many schools from different regions, and the repeated measurements of the sample. Unfortunately, it was not possible to arrange the measurements at all schools simultaneously. Nevertheless, the possible confounding effects of the differences in measurement time points among students allocated to the same group should have been eliminated in the analysis of the immediate effects, because all schools participated in the study with both experimental groups and control groups. When generalizing the results, it should also be borne in mind that with one exception, the intervention course was voluntary. The sample may not have been representative of students at all general upper secondary schools in that those volunteering to participate might have had different motives for taking the course than the other students.

Finally, the extensive baseline and post-intervention surveys in the study constituted both a strength and a limitation. On the one hand, they provided valuable information about the effects of the intervention, but on the other the students might have found them too exhausting to complete with care, which could explain the large drop-out numbers before the first baseline measurement (*n* = 67). Because of this, we shortened the follow-up measurement and did not include PANAS, PPS or CES-D as used in the baseline and post-intervention measurements. Nor was it possible to explore the long-term effects of the intervention using a control group on account of the waitlist study design. Therefore, the long-term effects of the intervention, not least on the PANAS and the PPS, remain unexplored.

### 5.2 Conclusions and future directions

The current findings indicate that the Study with Strength intervention course does enhance specific aspects of student well-being, including positive practices at school, positive emotions, and happiness. However, it does not have an impact on their general well-being or ill-being, including on symptoms of depression and study-related burnout. Nevertheless, the students reported a positive effect of the intervention on their personal lives and their studies. Overall, it appears that it had a small but positive impact, nudging the students towards enhanced well-being. Furthermore, the results offer valuable insights into the implementation of positive education on students at general upper secondary school aged 15–19.

To find out whether the intervention can be regarded as a protective factor for mental health, a deepened understanding of the reciprocal relationship between the effects of the intervention on well-and ill-being must be obtained. It would also be valuable to explore the effects of the intervention on other aspects of ill-being, such as stress or anxiety. Given the significant effect of the COVID-19 pandemic on the implementation of the Study with Strength intervention, replication would be of great value. Additionally, it would be useful to investigate the long-term effects of the intervention using a control group and a longer follow-up period, or to explore possible subgroups benefitting the most from the intervention. More studies using the PPS in school settings are also needed. Finally, it would be of significant value to find out how to further increase the effectiveness of the intervention through different implementation strategies, such as by implementing the Study with Strength intervention as part of a whole-school approach.

## Data availability statement

The datasets presented in this article are not readily available because of privacy issues. Requests to access the datasets should be directed to the corresponding author.

## Ethics statement

The study was approved by University of Helsinki Ethical Review Board and conducted in accordance with the local legislation and institutional requirements. Written consent was obtained from all participating students before the study. All students had turned 15 prior to the study, thus parental consent was not required according to Finnish legislation. However, the parents of the students taking the course were informed about the study.

## Author contributions

SF-W: Writing – original draft. ÅF: Writing – review & editing. MoL: Writing – review & editing. MaL: Writing – review & editing.
